# Antibacterial and Antibiofilm Activity of Carvacrol against Oral Pathogenic Bacteria

**DOI:** 10.3390/metabo12121255

**Published:** 2022-12-13

**Authors:** Irene Fernández-Babiano, María Luisa Navarro-Pérez, Ciro Pérez-Giraldo, María Coronada Fernández-Calderón

**Affiliations:** 1 Department of Biomedical Science, Area of Microbiology, University of Extremadura, 06006 Badajoz, Spain; 2 University Institute of Extremadura Sanity Research (INUBE), 06006 Badajoz, Spain; 3 Biomedical Research Network Centre on Bioengineering, Biomaterials and Nanomedicine (CIBER-BBN), 06006 Badajoz, Spain

**Keywords:** *Streptococcus*, carvacrol, antibacterial activity, biofilm, oral diseases

## Abstract

Faced with the current situation of high rates of microbial resistance, together with the scarcity of new antibiotics, it is necessary to search for and identify new antimicrobials, preferably natural, to alleviate this situation. The aim of this work was to evaluate the antibacterial activity of carvacrol (CAR), a phenolic compound of essential oils, against pathogenic microorganisms causing oral infections, such as *Streptococcus mutans* and *S. sanguinis*, never evaluated before. The minimum inhibitory and the minimum bactericidal concentration were 93.4 μg/mL and 373.6 μg/mL, respectively, for the two strains. The growth kinetics under different concentrations of CAR, as well as the bactericidal power were determined. The subinhibitory concentrations delayed and decreased bacterial growth. Its efficacy on mature biofilms was also tested. Finally, the possible hemolytic effect of CAR, not observable at the bactericidal concentrations under study, was evaluated. Findings obtained point to CAR as an excellent alternative agent to safely prevent periodontal diseases. In addition, it is important to highlight the use of an experimental methodology that includes dual-species biofilm and subinhibitory concentration models to determine optimal CAR treatment concentrations. Thus, CAR could be used preventively in mouthwashes or biomaterials, or in treatments to avoid existing antibiotic resistance.

## 1. Introduction

One of the most diverse and numerous microbiomes in the human body is found in the oral cavity, which is a complex environment harboring abundant nutrients, with a high level of humidity and a moderate temperature. In consequence, complex communities of microorganisms are found to co-exist there [1]. The majority of these are essential, however, and an imbalance can lead to oral diseases, such as periodontitis [2]. Furthermore, the bacteria in the oral cavity are not located in an arbitrary, isolated way, but rather occupy a determined place and have a specific function [3] according to a pattern of succession [4]. 

Within oral microbiota, the *Streptococcus* species represent between 60–80% of all primary colonizers [3] as they possess mechanisms to adhere to the salivary film, and are able to metabolize salivary components. Streptococci are important for the spatial and temporal development of oral biofilms, as well as their maintenance [1], and in particular *Streptococcus mutans* and *Streptococcus sanguinis*. *S. mutans* is one of the principal etiological agents in the formation of caries, and its action is mediated by both dependent and independent mechanisms of sucrose [5]. Moreover, it is particularly effective in the formation of biofilm in the hard tissues of the oral cavity. In addition to *S. mutans*, in other cariogenic bacteria, such as *Lactobacilli,* high levels of *S. salivarius*, *S. sobrinus*, and *S. parasanguinis* or *Veillonella* are found [6]. *S*. *sanguinis,* on the other hand, is a commensal bacteria in the oral biota which is, in principal, associated with good dental health; it is one of the main producers of H_2_O_2_, and helps to maintain oral symbiotic homeostasis [7]. The adhesion of *S. sanguinis* to dental surfaces is the first step in the formation of biofilm [8]. However, as a pioneer colonizer, *S. sanguinis* may also facilitate the attachment of subsequent pathogens. In addition, certain environmental conditions appear to affect the ability of *S. sanguinis* to maintain an ecologically balanced biofilm in the oral cavity [9]. Both species compete for colonization sites on dental surfaces [10] and help to bind other microorganisms in the later stages of biofilm formation. These two species, therefore, play a major role in the formation of oral biofilm. 

Infections caused by microbial biofilms are an issue of increasing importance in medicine. According to the National Institutes of Health (NIH), biofilm is involved in 80% of human bacterial infections [5]. Biofilms are complex, well-organized communities of microorganisms which secrete a matrix formed by exopolysaccharides that enables them to adhere to surfaces [11]. 

The tolerance and resistance of bacterial biofilms are factors which contribute to the difficulties faced by current therapies in treating infections associated to biofilms. This, combined with the fact that all currently available antibiotics were selected for their capacity to eliminate planktonic bacteria, as well as the situation of antibiotic abuse which has arisen in recent years, means that we are faced with the problem of bacterial resistance [12]. A wide range of strategies are being developed to avoid these problems, including novel antibiotics, combination and adjuvants of these, antiseptics and disinfectants, small molecule anti-biofilm agents, bacteriophage therapy, surface treatment and nanostructure functionalization, between others [13]. Recently, versatile and novel combination of techniques using photodynamic, photothermal, and gas therapy are proving to be a safe, multi-mode, synergistic antibacterial strategy for combatting biofilm infections [14,15].

More specifically, oral bacterial infections associated to biofilms constitute one of the principal causes of failure in implants, since the biomaterials used can enhance microbial adherence and colonization of the surgical device [16]. Furthermore, the secretions of mucopolysaccharides which cover and protect the bacteria make the infections difficult to eradicate. There is even evidence linking oral bacteria to systemic conditions, such as cardiovascular or respiratory illnesses, cerebrovascular accidents, premature births, diabetes, cancer, or pneumonia [1]. For all these reasons, it is surprising that despite the impact that biofilm-associated oral diseases cause in human health, sufficient strategies or more effective agents to combat these diseases have not been devised.

So far, several strategies have been developed that include antimicrobial dental materials based on the release of antimicrobial agents, contact killing, or combined strategies. In some cases, abrasive agents or mechanical removal of dental plaque are used. Probiotics, prebiotics or postbiotics has also been proved. Even studies involving enzymes and proteins are emerging [1]. An interesting option which has recently been gaining favor is the use of natural products, which can limit the onset and spread of these microorganisms effectively, for their use as substitutes or complements of current synthetic antibiotics [17,18]. Essential oils in particular have been shown to be powerful agents in treating infections and are safe for human and animal health [19]. These oils generally have a wide spectrum of bioactivity due to the presence in their chemical composition of various active functional groups (e.g., terpenes and aromatic and aliphatic components derived from phenol) [20]. 

An example is oregano oil, one of whose principal phenolic components, carvacrol (CAR) [2-metil-5- (1-metiletil) phenol], possesses multiple biological properties: anti-inflammatory [21]; antiparasitic [22], antimicrobial [17], antioxidant [23], hepatoprotective [22], and antitumoral [24]. In addition, some studies have shown that carvacrol inhibits bacterial biofilm formation [17], and its activity has been demonstrated in bacteria (*Escherichia coli*, *Staphylococcus aureus*, *Enterococcus faecalis*, *Salmonella*, *Gardnerella vaginalis*.), in yeast (*Candida* spp.), and in protozoans (*Giardia*, *Trypanosoma brucei).* Other in vitro investigations show that carvacrol has a selectively greater antimicrobial activity against pathogen bacteria than it has against beneficial probiotics [25]. However, it was not tested against the combination of two important oral strains.

For all these reasons, and given that CAR is a compound catalogued as GRAS (generally recognized as safe) [19] by the Food and Drugs Administration (FAD) of the USA [26], the aim of this study is to evaluate the antibacterial capacity of CAR against oral bacteria *S. mutans* and *S. sanguinis* for the possible application of carvacrol as an antimicrobial agent in preventing and controlling colonization by pathogen microorganisms causing oral infections in humans. 

## 2. Material and Methods

### 2.1. Preparation of Carvacrol Samples

Carvacrol (CAR), with a purity greater than 98% (0.956 g/mL), was purchased from Sigma (Sigma, St. Louis, MO, USA). The stock solution for all experiments was made by dissolving 1/10 in dimethyl sulfoxide (DMSO) solvent, and sterilized with 0.45 μm syringe filters (Millipore, Merck, Darmstandt, Germany). The study concentrations were determined as a percentage of this stock solution of CAR (95.6 mg/mL = 100%) and were from 12.5% (11.95 mg/mL) to 0.003% (2.9 µg/mL) in Trypticase Soy Broth (TSB, BBL™, BD, Becton, Dickinson and Company, Sparks, NV, USA) enriched with 1% sucrose (Panreac Quimica SLU, Barcelona, Spain).

### 2.2. Microorganisms and Culture Conditions

The strains included in this study were *Streptococcus mutans* ATCC 25175 and *Streptococcus sanguinis* ATCC 10556 (American Type Culture Collection). Bacteria were inoculated on Brain Heart Infusion Agar plates (BHA; OXOID, Ltd., Basingstoke, Hampshire, UK), and cultivated from independent colonies in tubes with 5 mL of trypticase soy broth (TSB) enriched with 1% sucrose (Panreac, AppliChem GmbH Ottoweg, Darmstadt, Germany). These cultures were incubated in an atmosphere supplemented with 5% CO_2_ for 12–18 h at 37 °C. After incubation, the inoculum was adjusted to 82% of transmittance at 492 nm in a vertical spectrophotometer (Helios epsilon Model, Thermo spectronic, Waltham, MA, USA), and diluted 1/100 in enriched TSB to obtain a bacterial suspension of approximately 10^6^ colony forming units per milliliter (CFU/mL). 

### 2.3. Antibacterial Activity 

Minimum inhibitory concentration (MIC) and minimum bactericidal concentration (MBC) were determined by the microdilution method in 96-well flat-bottomed polystyrene microtiter plates (Greiner bio-one, Frickenhausen, Germany) according to the Clinical and Laboratory Standards Institute guidelines [27], in flat-bottom microtiter plates. 

Each well contained a final volume of 200 μL, with 100 μL of the different CAR concentrations and 100 μL of the bacterial suspension prepared as indicated above. In addition, positive (no CAR) and negative (no bacteria) controls were included for each strain. The microplates were incubated in an aerobic atmosphere at 5% CO_2_ for 24 h at 37 °C. The MIC was then determined, both visually and by spectrophotometry (ELx800; Bio-Tek Instruments, Inc. Winooski, VT, USA), considered as the lowest concentration of antimicrobial able to inhibit the visible growth of the microorganism (O.D. ≤ 0.09) after a standard incubation time.

MBC, defined as the concentration capable of killing at least 99% of the initial inoculum, was also determined. For this purpose, 20 μL were taken from the wells in which no growth was visually observed, plated on BHI agar, and subsequently incubated at 37 °C and 5% CO_2_ for 24–48 h. After this time, the lowest concentration plate with no growth was considered as MBC. 

### 2.4. Growth Kinetics and Effect of Subinhibitory Concentrations on Biofilm Formation

Microbial growth evaluation was carried out in 96-well microtiter plates, under the same conditions as described above. Thus, 100 μL of CAR at different concentrations and 100 μL of bacterial inoculum were added to each well. Optical density was measured at 492 nm by a spectrophotometer (ELX800, Bio-Tek Instruments, Inc. Winooski, VT, USA) from the start the assay (T_0_) to 24 h (T_24_) at regular intervals of time.

For this analysis, the microdilution plate method was used following the protocol described above. The plates were incubated with 5% CO_2_ at 37 °C with orbital shaking (50 rpm) for 24 h. After the incubation time, the optical density was measured at 492 nm with a spectrophotometer to determine bacterial growth (planktonic plus biofilm). Later, planktonic bacteria were removed from the wells and gently washed twice with 200 μL PBS using a suction pump (Model FTA-2i, Biosan SIA. Riga, Latvia) to remove non-adhering bacteria. The biofilms adhering to the bottom of the wells were heat fixed, in a Pasteur Heraeus electronic oven (C.R. Maré, S.A., Barcelona, Spain), and then stained with violet crystal (VC, Gram-Hucker DC; Panreac, Barcelona Spain) for 5 min. Excess of colorant was removed with water. The plates were dried, and the colorant bound to the dyed cells was extracted with glacial acetic acid (GAA, Fisher Scientific, Loughborough, UK) at 33% for 10 min. Biofilm formation was quantified by measuring the absorbance of the GAA solution at 492 nm in a spectrophotometer. 

In order to determine whether the decrease in biofilm production was due to a reduction in bacterial growth by the different concentrations of CAR, the “slime index” (SI) was calculated according to the equation [28]:

SI% = 100 × [(mean O.D. of biofilm treated with CAR/mean O.D. of growth treated with CAR)/(mean O.D. of biofilm control/mean O.D. of growth control)].

The viability of planktonic and biofilm bacteria was evaluated using syto 9 and propidium iodide staining. Images representative was obtained by epifluorescence microscopy (Leitz DIAPLAN, Wetzlar, Germany) after kit Live/Dead Baclight Bacterial Viability L-7012 (Invitrogen SA. Eugene, OR, USA) staining.

### 2.5. Lethality Kinetics

To determine the bactericidal potential of CAR, lethality curves were made in tubes round bottom glass (0.6 × 100 mm). A 12-tube battery was prepared with 5 mL of enriched TSB and with 10^6^ CFU/mL of inoculum. The tubes were incubated at 37 °C in the presence of CO_2_ until the end of the exponential phase (approximately 12 h). An initial bacterial count (T_0_) was performed for each experimental condition. Then, the corresponding CAR concentration was added (without CAR, with 10× MIC or 100× MIC). 

Subsequently, bacterial counts were performed after 1, 4, and 24 h of incubation with the different experimental conditions in order to evaluate the variation of viable bacteria (planktonic and sessile) with respect to the control without CAR. 

Planktonic bacteria were measured after agitation, and an aliquot of the culture obtained, to perform a viable cell count. Adhering bacteria were separated by intense sonication for 5 min, and an aliquot was collected from the same tube as the planktonic ones to obtain the total number of bacteria. Bacterial recount was determined by serial dilutions and culture in BHA plates. The plates were incubated at 37 °C in the presence of CO_2_ for 24 h to obtain the CFU/mL. 

### 2.6. Activity on Mature Biofilms

The effect of CAR on mature biofilms was determined by measuring the metabolic activity of the bacteria within the mature biofilms. The assay was carried out on 96-well white polystyrene flat-bottomed microtiter plates (Greiner Bio-One, Frickenhausen, Germany). Hence, 200 μL of bacterial inoculum in TSB (10^7^ CFU/mL) of *S. mutans* or *S. sanguinis* or a mixture of both strains (100 μL of *S. mutans* + 100 μL of *S. sanguinis*) was added. The plates were incubated in 5% CO_2_ for 24 h at 37 °C for biofilm formation. Then, the microplates were washed twice with PBS to eliminate the non-adhering bacteria and the different concentrations of CAR (MIC, 2× MIC and 10× MIC) were added. The metabolic activity of the bacteria included in the biofilm was measured at 1, 4, 8, and 24 h of contact with the different CAR concentrations. 

The method used allows the determination of the number of viable microbial cells in biofilm cultures based on the quantification of ATP [29]. The BacTiter-Glo Microbial Cell Viability Assay (Promega Corporation, Madison, WI, USA) was conducted according to the manufacturer’s instructions. The reagents were added directly to the tested surfaces, allowed to act for 5 min in the dark with a gentle shake (20 rpm) and the light emission (luciferin-luciferase reaction) was measured using a fluorescence microplate reader (FLx800; Bio-Tek Instruments, Inc. Winooski, VT, USA). In the ATP-bioluminescence assays, the surfaces without bacteria were included as negative controls. 

### 2.7. Scanning Electron Microscopy of Biofilm

Biofilm was formed on Calgary device pins (Calgary Biofilm Device, CBD, Nunc, Thermo Scientific, Roskilde, Denmark), as a suitable tool for rapid and reproducible detection of the possible effects of antimicrobials on biofilms [30]. The CBD consists of two parts, a plastic lid that has 96 pins with an approximate area of 108.9 mm^2^ each, where the biofilm is formed; and a 96-well flat-bottom microtiter plate, which is used to deposit the bacterial inoculum. For this assay, an inoculum adjusted to 62% transmittance at a wavelength of 492 nm (approximately 10^7^ CFU/mL) was used. To the wells, 150 μL of the bacterial suspension in TSB was added and subsequently incubated for 24 h at 37 °C, 5% CO_2_ and shaking. After the first 24 h, the part of the device containing the pins was rinsed with 150 μL sterile PBS for about 10 s, and then transferred to a second microtiter plat containing 150 μL of different concentrations of CAR in the range from 12% to 0.006%. The pins were in contact with the different concentrations of CAR for an additional 24 h under the same incubation conditions as described above. To observe by scanning electron microscopy the biofilm morphology and the possible alteration produced by CAR, the pins were carefully washed twice with sterile TSBs to eliminate the non-adherent bacteria. The pins were removed from the lid of the Calgary device and were fixed at room temperature with 3% vol/vol glutaraldehyde (Panreac Quimica SAU, Barcelona, Spain) for approximately 18 h. Later, pins were washed with PBS for 5 min and dehydrated in a series of ethanol solutions (30, 50, 70, 90 and 100% vol/vol) for 1 h each. The samples were then dried in a vacuum chamber, coated with a thin layer of gold (≤5 nm) using an EMITECH K575K (Quorum Technologies Ltd., West Sussex, UK) sputter coater. Finally, the image was captured with a scanning electron microscope (Quanta 3D FEG, FEI Company, Hillsboro, OR, USA).

### 2.8. Influence on Erythrocytes

To evaluate the biocompatibility of CAR, its effect on erythrocytes was analyzed. An assay with discs impregnated with different concentrations of CAR was performed on sheep blood agar plates (OXOID LTD., Basingstoke, Hampshire, UK). Hemolytic damage was tested on plates cultured with *S. mutans* or *S. sanguinis* as well as on plates that did not have these strains. Plates were incubated with 5% CO_2_ for 24 or 48 h. Then, the presence of hemolysis halos in the plates produced by CAR was noted and measured. Negative controls were carried out with plates cultivated with *S. mutans* and *S. sanguinis* without CAR. 

Additionally, a study was performed in U-bottom microtiter plates (Greiner bio-one, Frickenhausen, Germany), using defibrinated horse blood (OXOID Ltd., Basingstoke, Hampshire, UK). In each well, 100 μL of different concentrations of CAR in PBS and 10 μL of blood were added. The assay was also performed adding 10 μL of bacteria to each of the wells. The negative controls were: PBS + blood or PBS + blood + bacteria. To ensure that the effect was not caused by the solvent, the same assay with DMSO also was performed.

To determine the percentage of hemolysis, 100 μL of red blood cells a final concentration of 4% (*v*/*v*) in PBS was deposited into a 96-well plate containing 100 μL of CAR (0–11.95 mg/mL) or 100 μL of 0.1% Triton-X100 as control positive [31]. PBS was used as a negative control, and the DMSO in which the CAR was dissolved was also evaluated. After incubation for 1 h, the supernatant (100 μL) was transferred to a new 96-well plate to record the absorbance at 540 nm. Percentage was calculated: hemolysis (%) = (Abs_540_ treatment CAR − Abs_540_ PBS/Abs_540_ Triton-X100 − Abs_540_ PBS) × 100%.

### 2.9. Statistical Analysis

All assays in this work were carried out in duplicate and repeated at least three times with independent cultures in order to confirm reproducibility.

The data obtained for the effect of sub-MIC on biofilm formation as IS was statistically analyzed using one-way analysis of variance (ANOVA) (Statistical Package IBM Statistics, SPSS v22.0; Chicago, IL, USA). The data are presented as mean ± SD of independent experiments. Differences were considered statistically significant at *p* values < 0.05. 

## 3. Results and Discussion

### 3.1. Determination of MICs and MBCs

MIC values for *S. mutans* and for *S. sanguinis* were found both visually and spectrometrically in the concentration 0.1% (93.4 μg/mL), while the MBC value corresponded to 0.39% (373.4 μg/mL). CAR is a lipophilic and water insoluble substance [17], so it was initially dissolved in an organic solvent such as DMSO. Before that, it was verified that this solvent produced no antimicrobial effect at the concentrations used in this study. Other authors who used ethanol as a solvent reported that it did influence CAR activity against, e.g., the flagellar motility of *E. coli* [32]. 

The essential oils that contain CAR have shown antimicrobial activity against both Gram-positive and Gram-negative bacteria [17]. The MIC ranges found for CAR vary between 250 and 2500 µg/mL depending on the microorganism and the experimental conditions [33]. Our results against *Streptococcus* spp, compared with other results for essential oils indicate that CAR has good activity against both bacteria [34]. Few studies report activity at lower concentrations: 32 µg/mL for the filamentous fungus *Trichophyton rubrum* and 128 µg/mL for *S. aureus* [35], 64–256 µg/mL for isolates of Group A *Streptococci* [36], and 125 μg/mL for *Streptococcus pyogenes* [37]. Some studies have shown the efficacy of this compound against resistant species, such as *Staphylococcus aureus* and *Salmonella* spp. [38,39], with MIC values between 14,550 and 29,100 μg/mL (approximately 12.5–25% of our concentrations).

With regard to bacteria found in the oral cavity, some authors have demonstrated total growth inhibition of *S. mutans* with 250 μg/mL of CAR [40]. Other authors, however, found a higher values, e.g., 0.25% (*v*/*v*) for *S. mutans* 0.25% (*v*/*v*) [41]; 400 µg/mL [42]; 250 µg/mL [43]; in this latter case the difference may be due to the fact that these authors obtained CAR by isolating it directly from essential oil of oregano. For *S. sanguinis,* MIC values of 125 μg/mL have been found [44]. However, in other cases, MIC values were at concentrations 20 times higher (2500 μg/mL) for both *S. sanguinis* and for *S. mutans* [45].

MBC ranges are also wide, from 112.5 to 900 μg/mL [46], or from 750 to 1530 μg/mL [47] for various organisms. In the case of *S. mutans*, MBC values of 0.5% (*v*/*v*) [41] and 600 μg/mL [42] have been found, and even 5000 μg/mL for *S. mutans* and *S. sanguinis* [45].

### 3.2. Effect of Sub-Inhibitory Concentrations

#### 3.2.1. Growth Curves

**Figure 1:**
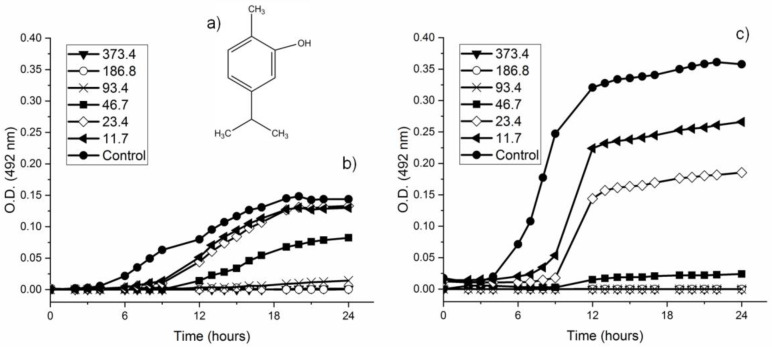
Chemical structure of CAR (**a**); growth curves of *S. mutans* (**b**) and *S. sanguinis* (**c**) against different concentrations of CAR (µg/mL) and Control (no treatment).

The study of bacterial inhibition through growth kinetics is a very useful indication of the antimicrobial action of the tested product. Figure 1 shows the growth curves for both strains (based on the O.D. 492 nm of bacterial cultures), without treatment and in the presence of different concentrations of CAR. As can be observed, the latency phase of the untreated culture (control) lasts approximately 4 h in both strains. The exponential or logarithmic phase lasts until 17 h for *S. mutans*, and until 12 h for *S. sanguinis*. The stationary phase lasted up to 24 h of incubation, the final point of our study. These growth phases, however, are clearly influenced when the bacteria are in contact with sub-MIC concentrations of CAR. In particular, it was observed that with higher concentrations of CAR to which the bacteria were exposed, from 0.01% (11.7 μg/mL) to 0.39% (373.4 μg/mL), the latency phases were more prolonged, the growth slopes were lower, and growth speed diminished, with the result that the maximum growth values of both untreated strains were not reached in any case. Specifically, in the case of *S. sanguinis*, the effects observed were more drastic, with delays in the latency phase, maximum growth rates, and bacterial concentrations being clearly evident. 

Few trials have studied this process exhaustively in oral strains of *Streptococcus*. In one assay with *S. mutans*, it was observed that, with a concentration of 100 μg/mL of CAR, a delay occurred in the logarithmic phase of the growth curve and higher concentrations did not allow the formation of the growth curve [42]. For other microorganisms, such as *Bacillus cereus,* it was reported that exposure of vegetative cells to concentrations of CAR of even 28.54 μg/mL led to an extension of the latency phase, a lower maximum specific growth rate, and a lower final population density, whereas at higher concentrations viability decreased exponentially [48]. The same tendency has been observed for *E. coli* [49] and for *S. aureus* [50]. 

#### 3.2.2. Biofilm Formation

**Figure 2:**
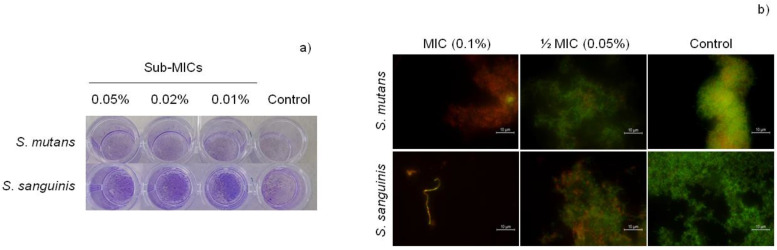
Biofilm remaining after treatment of CAR against *S. mutans* and *S. sanguinis* by violet crystal stain assay (**a**); Live/Dead staining of biofilm cells after treatment with CAR (**b**).

Figure 2 shows biofilm remaining after treatment with CAR stained with crystal violet (a) and live/dead assay (b). It can be seen how at sub-MIC concentrations there is an increase in biofilm biomass, although many of these bacteria are damaged. 

The influence of sub-MIC concentrations of CAR on biofilm production was evaluated by means of the “slime index”, shown in Table 1. It has been observed that the sub-MIC concentrations of CAR do not significantly influence biofilm formation of the two strains. The only significant difference (*p* less than 0.05) was observed with 0.25 sub-MIC of *S. sanguinis*, which indicates that in this specific case, the formation of biofilm is greater than expected based on the bacterial growth produced. In another study, biofilm of *S. mutans* was diminished with a sub-inhibitory concentration of 100 μg/mL [43]. This value corresponds approximately to the value that we determined as MIC. Experimental differences exist, such as culture medium, supplementation, or volume used, which would account for discrepancy between the results obtained. In the case of *S. pyogenes*, CAR inhibited biofilm formation in a concentration-dependent manner [51].

It is important to know the effect of sub-inhibitory concentrations both in bacterial growth and in biofilm formation, since exposure to sub-lethal concentrations of determined antimicrobial substances can produce greater resistance to the antimicrobial compounds [52]. 

### 3.3. Bacterial Lethality Curves

**Figure 3:**
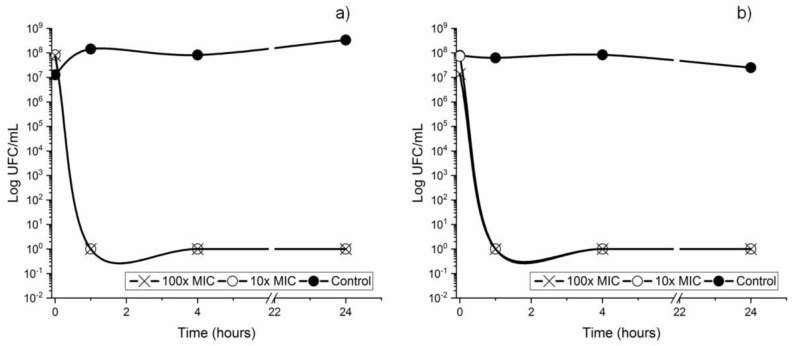
Time-kill curves for a standardized culture of *S. mutans* (**a**) and *S. sanguinis* (**b**) subjected to 10, 100 times their MIC and Control (no treatment) at different times.

Figure 3 shows the bacterial counts in UFC/mL of the bacterial culture in the exponential phase, after contact with CAR at a concentration of 10× MIC and 100× MIC (0.93 and 9.34 mg/mL 9, respectively). It has been observed that the bacterial capacity of CAR for both strains is very intense, since 100% of the culture bacteria is eliminated in the first hour. In agreement with other studies, CAR is a good bactericidal agent and affects both planktonic cells and cells which are beginning to form biofilm [53]. Until now, the lethality of CAR was only observed in *S. pyogenes*, for which 2× MIC (250 μg/mL) demonstrated an instant bactericidal action [51]. Lethality of CAR also was observed for gram negatives such as *E. coli*, in which concentrations of 1.5× MIC destroyed 95% of the bacteria in 10 min, and higher concentrations (3× and 4× MIC) totally eliminated the organisms in only 5 min [50]. 

### 3.4. CAR Activity in Mature Biofilms

**Figure 4:**
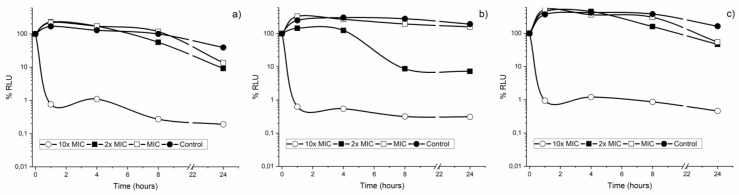
Variation of metabolic activity of the *S. mutans* (**a**), *S. sanguinis* (**b**) and mixture of both strains (**c**) included in mature biofilm under various concentrations of CAR and Control (no treatment) at different times.

One of the effects of CAR seems to be its capacity to inhibit activity of ATPases [54], and therefore would have an influence on the amount of ATP. The effect of CAR on mature biofilms is represented in Figure 4. The graph shows variation of cell metabolic activity through the amount of intercellular ATP present, measured in relative luminescent units (RLU). The percentage of bioluminescence of the two strains separately, *S. mutans* (a) and *S. sanguinis* (b), and together (c) was measured at different concentrations and contact times with CAR (control, 2× MIC and 10× MIC). The concentration of 10× MIC diminished bacterial activity at 1 h of contact in the three cases (a, b, and c). For the rest of the concentrations, it was observed that the *S. sanguinis* biofilm is more sensitive to CAR than that of *S. mutans* or that formed by both together. Concentrations of 2× MIC were able to diminish almost by half metabolic activity of the *S. sanguinis* cells present in the biofilm after 1 h of contact. Another interesting fact is that with the concentrations which did not eradicate the biofilm, initially, bacterial activity is seen to increase, reaching higher values than the control, although with time this activity decreases. As with the results of the lethality curves, it was observed that concentrations of 10× MIC are necessary to eliminate rapidly and effectively the bacteria present in the biofilm, which is very important to avoid resistance. 

The amount of intracellular ATP was studied both in biofilms composed of a single bacterial species and in those formed by the mixture of the two study strains, with the aim of observing possible differences in the degree of impact produced by CAR in the biofilms, since natural biofilms are composed of several microbial species. Furthermore, in biofilms, some organisms are dependent on others, so the mixture of microorganisms in the study of biofilms can provide more authentic results regarding the behavior of these against the compound studied [55]. In general terms, we can state that, as CAR concentration and contact time increase, intracellular ATP decreases, regardless of the strain studied, and that when both strains grow together they form a biofilm with a greater metabolic activity at sub-lethal concentrations than when the strains grow separately. 

From these results we can appreciate the difference that exists between the MICs determined for planktonic bacteria and the concentration necessary to eradicate a pre-formed biofilm. The MIC determined in the previous section was ineffective in eradicating the already formed biofilm. In *S. pyogenes* also from 2× MIC (250 μg/mL) was observed eradication of preformed biofilms [51]. Other authors have reported for *Staphylococcus* that the concentrations of biofilm eradication are 2–4 times greater than those necessary to inhibit growth in suspension [56]. 

CAR activity is related with the presence of a hydroxide group its structure, which confers lipophilic properties, allowing the molecule to partition between the lipids of the cytoplasmic membrane and the mitochondria, disrupting the structures, thus increasing their permeability and causing the cellular contents to escape. In addition, the polarization and depolarization of the membrane together with the delocalized electron system that its structure contains also play an essential role in the antimicrobial activity. CAR functions as monovalent cation transmembrane transporter by interchanging its H^+^ proton for a K^+^ ion between the cellular cytoplasm and the exterior [57,58]. This results in the absence of a proton motor force and, consequently, ATP cannot be further synthesized. Depletion of ATP reserves leads to a deterioration of the essential processes in the cell. CAR also interferes with nucleic acid metabolism [59]. Ultimately, all these events result in cell death. It has also been seen that CAR can prevent biofilm formation at the initial and subsequent stages due to its inhibition of metabolic activity [53]. Furthermore, it appears that the reduction in cell surface hydrophobicity by carvacrol observed for *S. pyogenes* also plays an essential role in the antimicrobial activity [51].

In sum, according to the relevant literature, CAR has a multiple action mechanism based on alteration of the cytoplasmic membrane, interruption of the flow of electrons, interference with active transport, and inhibition of coagulation of the cytoplasmic content and of enzyme activity. Based on our results, the increase in metabolic activity, measured as an increase in intracellular ATP, demonstrates that at sub-lethal concentrations to eliminate the biofilm (2 × MIC and MIC), the bacterial cells probably generate a “stress” response, during the first hours, that increases their activity in order to avoid the effect induced by CAR.

### 3.5. Ultramicroscopic Study of Biofilm

**Figure 5:**
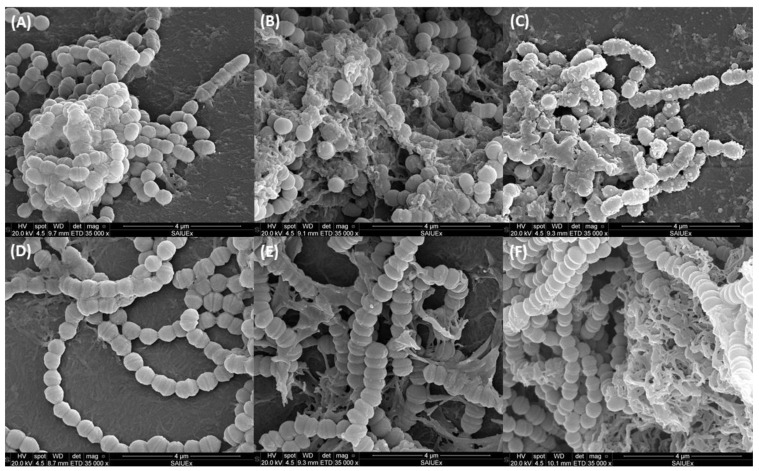
Image obtained by scanning electron microscopy of the biofilm generated by *S. mutans* (**above**) and *S. sanguinis* (**below**) at two different concentrations of CAR ((**A**,**D**): Control; (**B**,**E**): 1.5 mg/mL (1.5%), (**C**,**F**): 11.95 mg/mL(12.5%)).

Figure 5 shows the 1-day-old biofilms of *S. mutans* (A–C) and *S. sanguinis* (D–F) with subsequent treatment with CAR at bactericidal concentrations (1.5% and 12.5%) or TSB as control. After 24 h of contact was observed, in the control, bacteria forming chains and the construction of certain amorphous masses that are probably correlated to the intercellular polymeric substance. The cells are well preserved, with an approximate diameter of 0.7 μm, and have a smooth surface which is apparently healthy and with clearly differentiated division grooves. 

In the specific case of *S. mutans* (Figure 5B,C) it can be observed that, in face of the increased concentration of CAR, the intercellular substance is more abundant and its morphology is different from that of the control, with certain amorphous masses, probably of an intercellular polymeric substance which causes the cells to cohere among themselves. In particular, in the presence of 12.5% (11.95 mg/mL) CAR (Figure 5C), the bacteria appear deformed and in many cases their surface is no longer smooth. Additionally, vesicular conformations appear. Some authors state that these structures present on the outer surface of the cells represent a collection of cytoplasmic constituents which have been pushed through cracks in the cell wall [60]. 

In line with our results, some studies have observed the same effect: lessening of the bacterial chains and of cellular smoothness. In fact, analysis by atomic force microscopy confirmed that the outer surface of gram-positive and gram-negative bacteria was the main target of the compound [61]. In this study, size, length, and diameter of the cell decreased after contact with CAR. Some authors have analyzed the effect of CAR on *S. mutans* by TEM analysis and found that the cells treated with 1% CAR suffered alterations and breaks in the membranes [41]. SEM analysis has also shown deformation and smoothness of cells, and shorter chains than in the controls, which is in agreement with our results [43]. The images presented by these authors, however, show apparently clean bacteria, with little intercellular polymeric material. The methodology used may be the cause of these differences, since the Calgary device is used to prevent biofilm formation by deposition. As a consequence, the bacterial interconnections in the spicule may be increased, causing an enhanced of exopolysaccharide substance production. 

*S. sanguinis*, which in the control can be observed forming long chains with a relatively clean morphology, when incubated with CAR (Figure 5E,F), appears agglutinated in microcolonies of between 100 and 150 µm which include apparently well-structured chains of cocci. In this case, the extracellular amorphous substance also reappears, though in lesser quantity and without visible bacterial deformation. This difference found between both strains could be due to the fact that *S. mutans* produces a biofilm of exopolysaccharides which protects the bacteria from harmful conditions [62,63]. 

The action mechanism of CAR would seem to be multiple [58], and this depends to a great extent on the structural characteristics of the molecule, which presents a free hydroxide group, a delocalized system of electrons, and a hydrophobic character. One of its possible targets is the cytoplasmic membrane, altering both its development and its function. Given its hydrophobic nature, it interacts with the lipid biofilm of the cytoplasmic membrane and aligns between the chains of fatty acids, causing expansion and destabilization of its structure, increasing the fluidity and the permeability to potassium ions and protons, resulting in subsequent death of the cell [17]. This fact could be related with the morphological change observed in the images obtained by SEM. 

### 3.6. Influence of CAR on Erythrocytes

**Figure 6:**
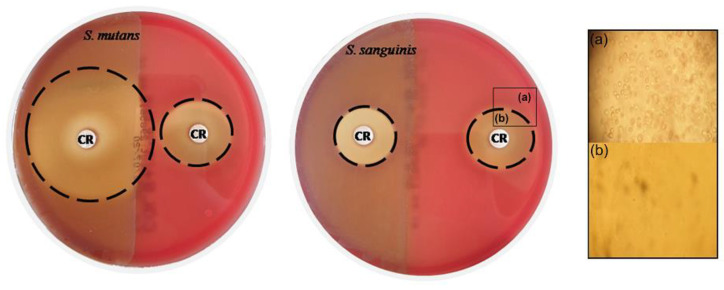
Hemolytic effect of CAR on blood agar plates. The left halves of both plates were inoculated with *S. mutans* (**left**) and *S. sanguinis* (**right**). (**a**) Healthy and (**b**) Hemolyzed red blood cells observed by optical microscopy. Disks contained CAR at 95.6 mg/mL.

Figure 6 shows how the disks impregnated with CAR produces a hemolysis halo evident at concentrations of 100% (95.6 mg/mL) of CAR (22–30 mm). This halo was considerably smaller at lower concentrations of 12.5% (11.95 mg/mL) (7 mm) and inexistent at the highest concentration, used in the other assays, of 1% (0.96 mg/mL) (data not shown). As can be observed (Figure 6b), the hemolysis produced causes total destruction of the red blood cells. 

When the hemolytic capacity of CAR was compared on previously inoculated plates and uninoculated plates with the two strains, it was seen that this hemolytic capacity is enhanced when *S. mutans* (α-hemolytic) is present on the plate, but no differences are appreciated when the plate of blood agar contains *S. sanguinis* (α-hemolytic), i.e., the hemolytic effect is stronger in the presence of *S. mutans*, which is an important finding if it is used with the aim of achieving asepsis of the oral cavity. 

**Figure 7:**
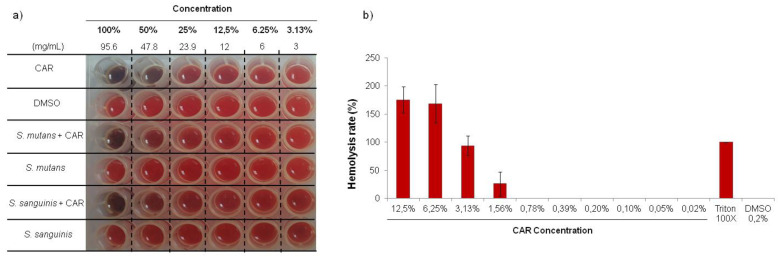
Hemolytic effect of different concentrations of CAR and solvent (DMSO) in the presence and absence of the strains studied in microtiter plate (**a**); Hemolysis rate (**b**).

In the assay on microtiter plates (Figure 7), the hemolysis only occurred at the highest concentrations (95.6–1.5 mg/mL), although visually it is only visible in the two highest concentrations (Figure 7a). The concentrations which were closest to MIC (0.1% = 93.4 µg/mL) showed no differences with respect to the negative controls as demonstrated by the hemolysis rate (Figure 7b). With regard to DMSO, there were no differences compared to the controls, and the bacteria alone did not appear to generate hemolysis.

The hemolytic effect produced by the presence of CAR, as previously mentioned, only appears at high concentrations, far from the values proposed as suitable for treatment against the two bacterial species. Furthermore, there are studies which value the safety and tolerance of CAR even at high doses (1 and 2 mg/kg/days) [64]. In this clinical assay, it was determined that CAR did not produce a negative effect on the health of the subjects who participated. On the contrary, CAR was seen to protect the ADN from damage and demonstrate antigenotoxic activity [17]. Although CAR has a low genotoxic potential even at high doses (105.154 µg/mL) [42], it has been found to have a cytotoxic effect against different cell lines of human cancer [65].

As a result of the appearance of antimicrobial resistance (AMR), the Center for Disease Control (CDC) has supported the crucial need to investigate new substances with the antimicrobial potential to combat resistant strains. Of these substances, essential oils represent an interesting option, but their complex composition, amongst other factors, makes them varied in their bactericidal responses. Thus, it is advisable to focus on their principal components, as in the present study where we focused on the use of CAR against oral bacteria. In view of the results, CAR can be used to introduce it in mouthwashes to maintain the balance of the oral microbiota. The biofilm formation associated with this type of infection is another important challenge, which means that findings of this work could promote the use of CAR for its treatment. For example, controlled systems using molecules capable of self-assembling into stable nanoparticles which incorporate diverse antibacterial compounds are being investigated currently [66,67]. Moreover, the incorporation of CAR in implantable and biodegradable biomaterials, either by loading or coating them with this substance to prevent the biofilm formation by these or other multidrug-resistant bacteria, could also be considered in future studies.

## 4. Conclusions

CAR presents suitable antimicrobial activity against dominant “pioneer” species in the oral cavity, such as *S. mutans* and *S. sanguinis*. The inhibitory and bactericidal concentrations are sufficiently low to propose this compound as a good candidate for formulations of daily oral hygiene. This study has shown that sub-inhibitory concentrations of CAR detain and considerably delay the growth of these species. In addition to this, the death kinetics shows a rapid and efficacious microbicide action at concentrations which affect planktonic bacteria in the same way as those protected in their polymeric matrix. Furthermore, the relevance of using a mature biofilm model to evaluate antibacterial activity and optimal eradication concentrations has been demonstrated. A rapid and effective antibiofilm action of CAR has been observed for both mono- and dual-species biofilm, not previously studied. The results of this work, together with other biological characteristics, point to CAR as a good alternative agent for use within traditional protocols to prevent periodontal diseases as an adjuvant in the treatment or assessment to include in biomaterials.

## Figures and Tables

**Table 1:** Percentaje of *Slime Index* values for sub-MIC concentrations of CAR.

Concentration CAR	*S. mutans*	*S. sanguinis*
½ MIC (0.05% = 46.7 µg/mL)	70.21% ± 43.81	189.52% ± 35.68
¼ MIC (0.02% = 23.3 µg/mL)	95.53% ± 19.01	143.75% ± 32.46 *
⅛ MIC (0.01% = 11.7 µg/mL)	114.35% ± 21.16	114.97% ± 50.58

Values (mean ± SD) were obtained from assays performed in duplicate and repeated three times. (*) Statistically significant differences (*p* < 0.05) with respect to the control values (100%).

## Data Availability

The data presented in this study are available in article.
